# Investigating hydrogel formation using *in situ* variable-temperature scanning probe microscopy†
†Electronic supplementary information (ESI) available: Additional scanning probe micrographs, cryo-TEM micrographs, NMR spectra, and crystal packing diagrams. CCDC 1406421. For ESI and crystallographic data in CIF or other electronic format see DOI: 10.1039/c5sc02196k


**DOI:** 10.1039/c5sc02196k

**Published:** 2015-08-03

**Authors:** Emily C. Barker, Ching Yong Goh, Franca Jones, Mauro Mocerino, Brian W. Skelton, Thomas Becker, Mark I. Ogden

**Affiliations:** a Department of Chemistry and Nanochemistry Research Institute , Curtin University , GPO Box U1987 , Perth , Western Australia 6845 , Australia . Email: t.becker@curtin.edu.au ; Email: m.ogden@curtin.edu.au; b Centre for Microscopy , Characterisation and Analysis , M310 , University of Western Australia , Perth , Western Australia 6009 , Australia

## Abstract

The assembly and disassembly of supramolecular gel fibres are observed *in situ* using variable temperature scanning probe microscopy. The results show that fibre formation can be monitored at high resolution at a surface, and the final fibre morphologies are broadly consistent with those found by *ex situ* analysis of the bulk gel. The impact of a gelation inhibitor upon the fibre morphology is successfully investigated, providing direct evidence for the mechanism of inhibition as a function of additive concentration.

## Abstract 2

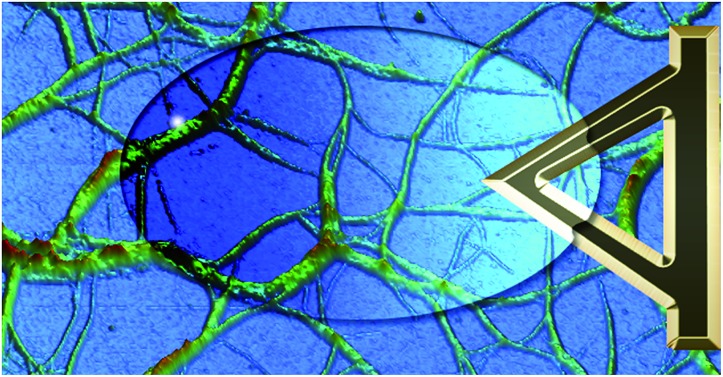
The assembly and disassembly of fibres formed by a low molecular weight hydrogelator are imaged at high resolution.

## Introduction

Supramolecular gels, formed through the assembly of low molecular weight gelators (LMWGs), have been known and used industrially for decades.^1^ They are attracting intense interest in recent years because of the potential applications that arise from their capacity to be “switched” rapidly between the gel and sol by an external stimulus,^2^ with excellent control of the gel properties.^3^ These potential applications, which include drug delivery, tissue engineering, smart materials, crystal growth control, and sensors,^1^,^4^ will be most efficiently developed if there is a robust understanding of the self-assembly and disassembly processes that take place during gel–sol transitions. To date, characterisation of gel structures has typically relied on imaging of dried gel samples using electron or scanning probe microscopy,^5^ or the typically lower resolution option of confocal laser microscopy of “wet” gels.^6^ A significant concern is the introduction of artefacts during drying processes, although it is possible to correlate microscopy results with X-ray or neutron scattering data to determine if dramatic changes have occurred.2*d* Functionalisation of gelators with fluorescent probes has also been used to provide indirect structural information.^7^ We have reported the use of scanning probe microscopy to image, while wet, smeared hydrogel samples on mica.^8^ Here, the unknown is the impact that the shear forces applied during sample preparation may be having on the gel fibre morphology.

What is missing from this array of analytical techniques is a method to image the assembly and disassembly of the solid-like network within the liquid phase as it occurs, a process that is central to many of the potential applications of these systems, as well as being required for fundamental understanding of self-assembled gels. To that end, we report here the use of PeakForce Tapping mode for variable temperature *in situ* imaging of gel fibre assembly and disassembly in a scanning probe microscope. This imaging mode provides highly accurate direct force control, with forces between the AFM probe and the sample in the order of pico- to nano-Newton. Unlike standard *in situ* Tapping Mode, PeakForce Tapping is not affected by temperature changes in the solution because the cantilever is driven at a frequency well below its resonance and thus is well suited for *in situ* investigation of gel formation processes.^9^ We note that this imaging mode has been used successfully to examine secondary structure changes in polysaccharide chains occurring slowly over time periods of up to 2 hours,^10^ but to our knowledge, high resolution imaging of LMWGs under dynamic conditions has not been reported. Such a technique would both complement and test the growing range of predictive structure–activity methods being developed for gel formation.^11^

**Fig. 1:**
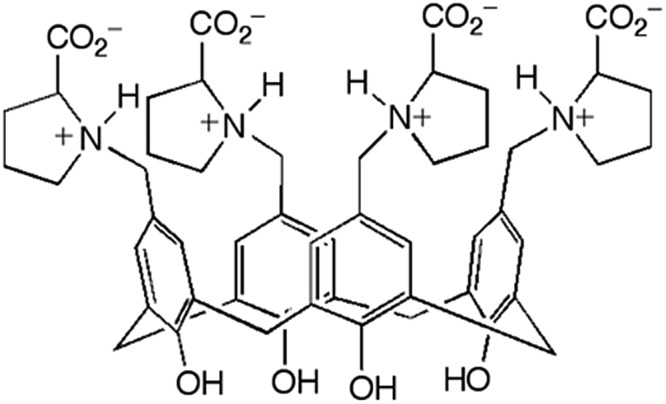
Proline-functionalised calix[4]arene, **1**, the low molecular weight hydrogelator used in this study.

Hydrogels formed by the proline-functionalised calixarene **1** were chosen for this study (Fig. 1).^8^ Gelation of **1** is triggered by the addition of a salt, and the strength and stability of the gel can be finely tuned by changing the concentration and composition of the added electrolyte. The gels are thermoreversible, and are typically transparent making them ideal for the development of this technique.

## Experimental

Proline calixarene **1** was synthesised according to the literature.8*a* Solvents and metal salts were used as supplied.

### *In situ* scanning probe microscopy

AFM imaging was performed using a Bruker Dimension FastScan AFM system operating in PeakForce Tapping mode. Unlike standard intermittent contact mode, PeakForce Tapping utilises a frequency far below the resonant frequency of the cantilever to drive the cantilever and is significantly less sensitive to changes in the scanning environment. The AFM experiments were performed in drops of solutions on freshly cleaved mica substrates mounted on a heating/cooling stage. The gelling solutions were formed by mixing an aqueous solution of **1** with a magnesium chloride solution (both typically in the 20–25 mM concentration range in the final solution; concentrations are specified in the Figure captions), which was then introduced to the sample stage using a Pasteur pipette before gel formation proceeded to any significant extent. Imaging commenced immediately after the injection of the solutions at room temperature. Once the tip is engaged with the surface the stage temperature was lowered or increased to initiate the formation of the gel or to disassemble gel fibres. If gel fibres were imaged immediately after loading the sample, typically the temperature was increased until no fibres were detected at the surface, after which the temperature was reduced until fibre formation was observed. The AFM images presented in this study were acquired with Bruker FastScan-B probes (spring constant 0.8 N m^–1^) at a peak force of 1–2 nN between probe and sample. The tip velocity or scan rate has to be determined based on how often the probe taps on the sample, *i.e.* the probe has to touch the sample at each imaging pixel during the PeakForce Tapping oscillation. The cantilever was driven at a frequency of 2 kHz in PeakForce Tapping mode. This allows for a maximum scan rate of 1.95 Hz at 512 × 512 data points per image or 3.9 Hz at 256 × 256 data points per image, resulting in shortest acquisition times of 4.4 min per image and 1.1 min per image. For reasons of scanner stability during data acquisition the scan rate was kept constant at 1.95 Hz. Due to the somewhat limited time resolution of PeakForce Tapping compared to standard Tapping Mode, the temperature variations to induce assembly and disassembly of the gel have to be performed in small steps. However, the ability of continuous scanning without the need for retuning the cantilever outweighs the limited time resolution in this study. The raw AFM data were processed using Plane Fitting within the Bruker Nanoscope Analysis 1.50 software.

### Crystallography

Crystals of **1** thf were grown by diffusion of thf vapours into an aqueous solution of **1**. The crystals immediately desolvated upon removal from the mother liquor, but if kept wet with solvent were of sufficient quality for single crystal X-ray structure determination.‡
‡Crystal data for **1** thf: Empirical formula C_56_H_68_N_4_O_13_; MW = 1005.14. Cubic, space group *F*432, *a* = 36.2659(2), volume = 47 697.5(8) Å^3^, *Z* = 24; *ρ*_c_ = 0.84 Mg m^–3^, *μ* = 0.489 mm^–1^, crystal size 0.58 × 0.33 × 0.24 mm^3^; *θ*_min,max_ = 3.45, 67.28°. Reflections collected = 144382, unique reflections = 3613 [*R*(int) = 0.058]. Max./min. transmission = 1.00/0.859. Number of parameters = 175. Number of restraints = 144, *S* = 1.323. Final *R* indices [*I* > 2*σ*(*I*)] *R*_1_ = 0.1157, w*R*_2_ = 0.3037; *R* indices (all data) *R*_1_ = 0.1253, w*R*_2_ = 0.3188. Largest diff. peak and hole 0.418 and –0.558 e Å^–3^.


Crystallographic data were collected at 150(2) K on an Oxford Diffraction Gemini diffractometer fitted with Cu Kα radiation. Following analytical absorption corrections and solution by direct methods, the structure was refined against *F*^2^ with full-matrix least-squares using the program SHELXL-2014.^12^ The solvent in the calix was modelled as thf disordered about the 4-fold axis of cubic space group *F*432. The non-hydrogen atoms of the thf molecule were refined with isotropic displacement parameters with geometries restrained to ideal values. Anisotropic displacement parameters were employed for the remaining non-hydrogen atoms. Hydrogen atoms were added at calculated positions and refined by use of a riding model with isotropic displacement parameters based on those of the parent atom. Attempts to model the remaining electron density as further thf molecules were unsuccessful. The program Squeeze was used to effectively remove any such electron density. The H atom was assumed to be on the calix phenol O atoms rather than one of the carboxylate O atoms due to possible H-bonding considerations.†

## Results and discussion

**Fig. 2:**
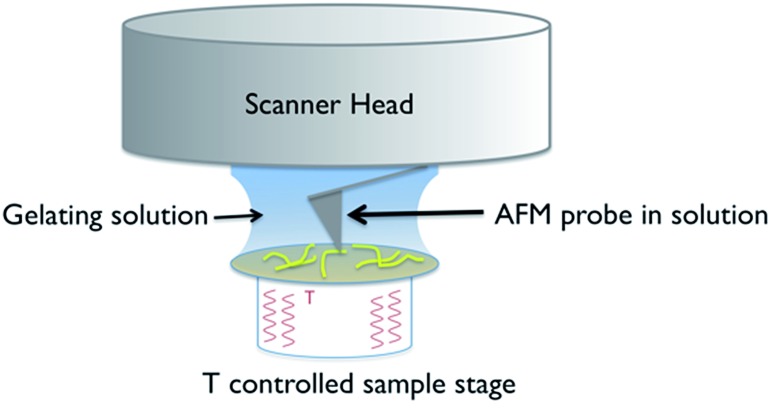
Schematic of the experimental set up used for *in situ* variable temperature scanning probe microscopy of gel formation.

A schematic of the experimental arrangement used is shown in Fig. 2.

**Fig. 3:**
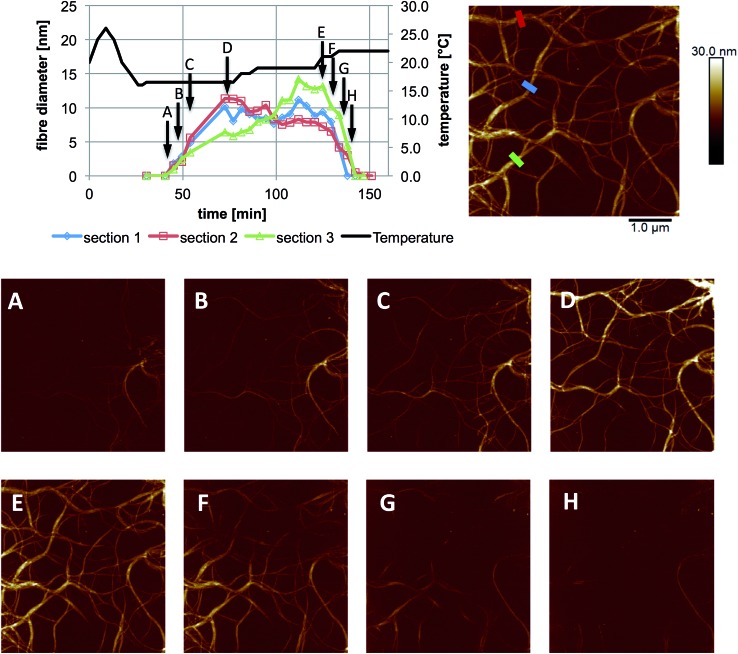
*In situ* images and cross sectional analysis of fibres formed by an aqueous mixture of **1** (25 mM) and MgCl_2_ (20 mM) with changing temperature. All cross sections were taken as an average over a length of 250 nm along the fibre. The height scale is 0 to 30 nm, and scan size is 5 μm, in all images; 4.4 min per image. The full set of images are provided in Fig. S1.†

The use of a solution cell is avoided by studying a hydrogel, as the water surface tension ensures that a droplet of the solution stays in place when introduced on to the mica substrate. The hydrophilic mica surface was chosen as the substrate, as hydrophobic interactions control the assembly of **1**, and thus a hydrophilic surface was deemed the least likely to disrupt gelation. The cantilever is immersed in the droplet, and the gelation process is controlled by altering the temperature of the sample stage. Cooling induces the formation of gel fibres, which are imaged at the mica surface (Fig. 3 and S1†). Over time, the density and thickness of the fibres both increase. Heating disassembles the fibres, and if continued for sufficient time leaves a clean mica surface. The cycle can be repeated until evaporation of the solvent becomes significant or the gel collapses due to crystallisation.

**Fig. 4:**
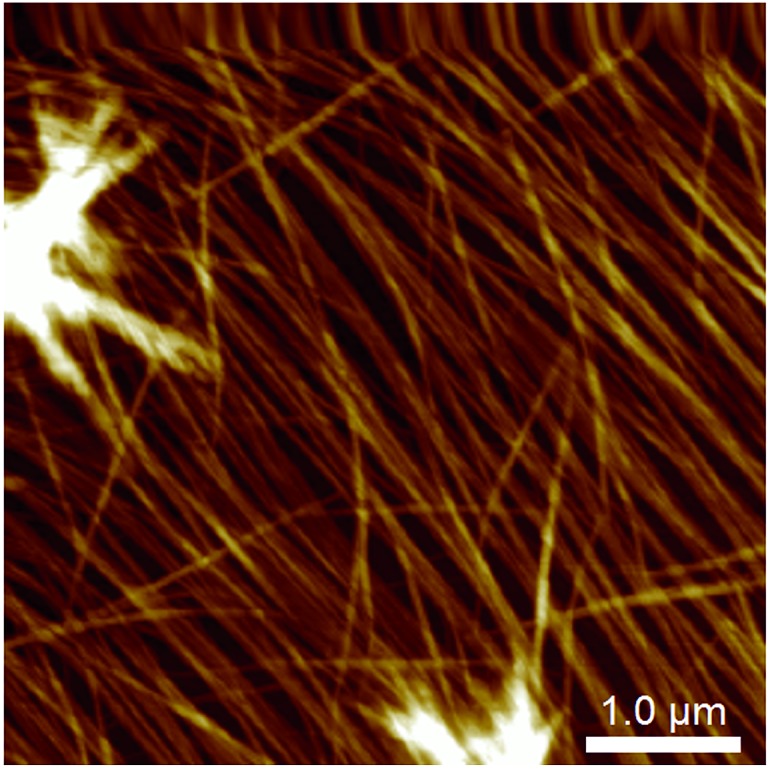
Atomic force micrograph of a **1**/MgCl_2_ hydrogel smeared and imaged *ex situ* on a mica substrate, showing alignment of the fibres presumed to be induced by the sample preparation.

These experiments capture, for the first time to our knowledge, the assembly and disassembly of LMWG fibres in real time. There are, however, a number of issues to address. The most obvious of these is the impact of imaging at a surface. This is an unavoidable aspect of the technique, and while surfaces have been elegantly used to control assembly processes,^13^ here the intent is to reflect the bulk structure as much as is possible. A comparison of the *in situ* images with those obtained by smearing a bulk sample of the gel on mica (Fig. 4) shows similar fibrous structures, aside from the alignment of fibres in the *ex situ* sample, which is ascribed to the shear forces applied during sample preparation. Repeated cycling of the temperature to assemble and disassemble the gel leads to differing distributions of fibres at the surface after each cycle (Fig. S2†). This suggests that the surface is not having a substantial effect on the fibre morphology. Another consideration is the influence of the surface as a possible nucleation site for fibre formation, leading to localised gelation at the surface. The bulk *T*_gel_ of the system used in Fig. 3 is ∼20 °C (as determined by a simple vial inversion test). Fibre formation *in situ* was initiated by reducing the temperature of the stage to 16 °C, and the fibres formed persisted as the temperature was increased to 19 °C (Fig. 3). Increasing the temperature to 22 °C caused the fibres to disassemble. These results are reasonably consistent with the bulk measurements, suggesting the surface is not having a large impact on the fibre formation processes. The density of the fibres observed at the surface was also qualitatively positively correlated with the extent of hysteresis in the PeakForce Tapping force curves.§
§It should be noted that a limitation of this technique is that if the hysteresis of the force curve becomes too extreme due to the strength of the gel, imaging can not continue. This is consistent with gel formation throughout the bulk of the droplet. Indeed, the tendency observed was that long fibres would appear at the surface apparently complete, suggesting that fibres already formed in the bulk liquid may be moving to the surface, rather than growth in length at the surface taking place.¶
¶CryoTEM imaging of samples from solutions of **1** with various metal salts at concentrations below that required for gel formation showed the presence of fibres, suggesting that these assemblies form readily in solution (Fig. S3†). Once at the surface, however, a growth process was clearly observed in that thickening of the fibres occurred over time, consistent with fibre bundling, as shown in Fig. 3. Heating to disassemble the gel did not lead to a direct reversal of these processes, with the fibres often being left as shorter segments with dissolution sites occurring along their length.

**Fig. 5:**
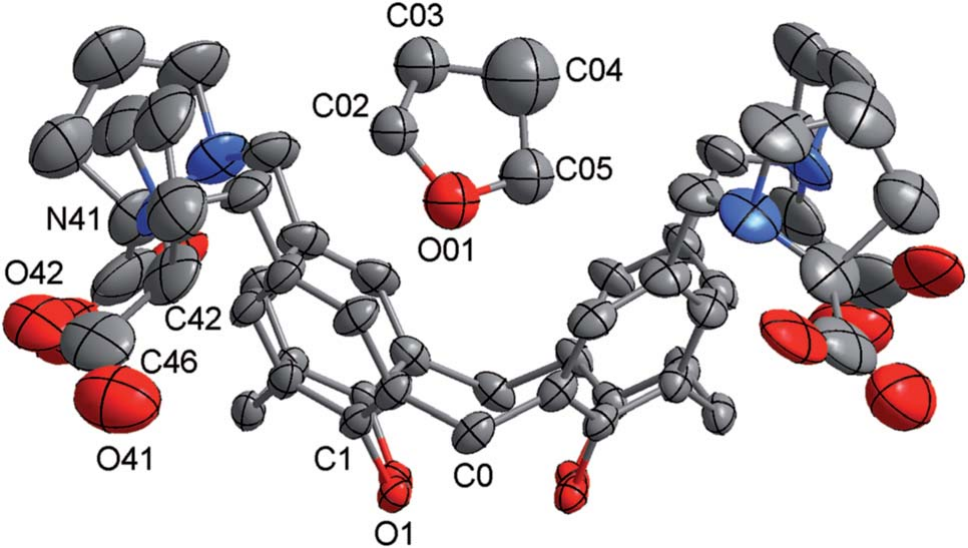
A representation of the structure of **1**·thf. Hydrogen atoms are omitted for clarity.

**Fig. 6:**
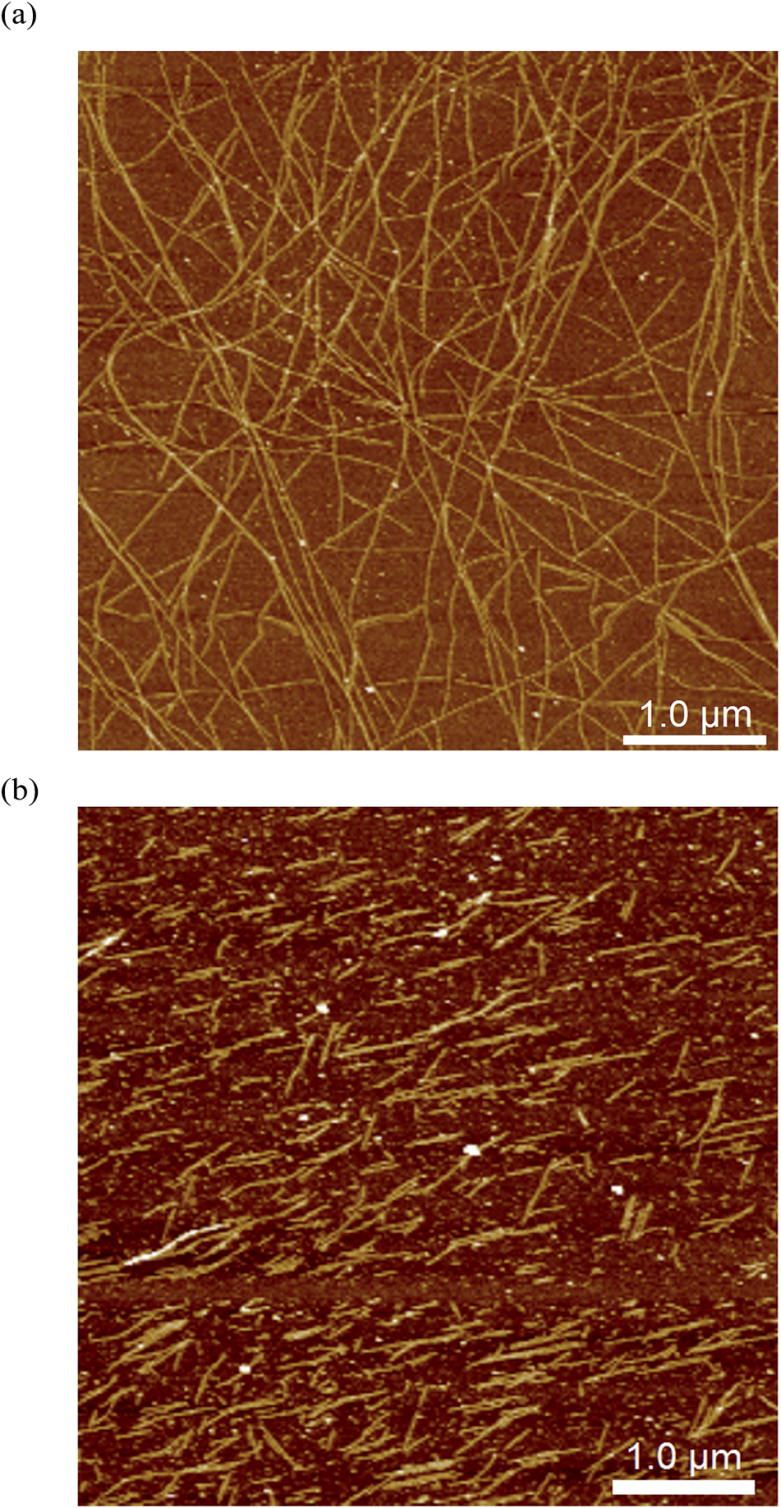
*In situ* images of fibres formed in an aqueous mixture of **1** (20 mM) and MgCl_2_ (25 mM) with addition of (a) 0.6% v/v thf, (b) 2% v/v thf, imaged at 22 °C.

The impact of additives or guests on gel formation and structure is important to understand in the context of potential applications. As a model system, the influence of tetrahydrofuran (thf) on the gelation behaviour of **1** was investigated. We have previously reported “self-inclusion” of a proline moiety in a neighbouring calixarene cavity as an interaction that could lead to high aspect ratio structures required for fibre formation.8*b* Guests that can compete with this interaction should therefore destabilise the gel. Proton NMR studies (Fig. S4†) show that thf can be included in **1** in solution. Structural analysis of crystals formed by diffusing thf into an aqueous solution of **1** confirmed that thf can act as a guest (Fig. 5 and S5†). Consistent with these results, addition of thf to a solution of **1** inhibits the tendency to form a gel when a salt is added. For example, a mixture of **1** (20 mM) and MgCl_2_ (25 mM) has a *T*_gel_ of 28 °C. In the presence of 0.6% v/v of thf no bulk gelation is observed down to 0 °C. Attempting to image these systems *ex situ* when drop-cast on mica gave inconsistent results, with dramatically different morphologies on different areas of the sample (Fig. S6†). This may have been due to restructuring as the more volatile thf evaporated. Examining the samples *in situ*, however, gave more consistent results and added some fascinating insight into the inhibition mechanism. Rather than shortening the gel fibres to prevent gelation, the thf additive in fact appeared to inhibit bundling of the fibres, with remarkably long single fibres observed despite the lack of bulk gelation (Fig. 6a). This behaviour would also lead to prevention of gel formation,^14^ but is not consistent with the original hypothesis that the gel fibres would shorten because of competitive inclusion. Upon increasing the thf concentration to 2% v/v, shorter fibres were observed (Fig. 6b), but overall the *in situ* results suggest reduction of bundling is the initial, and perhaps dominant, cause of the gel inhibition. Cycling the temperature once again enabled disassembly and reassembly of the fibres to be observed, but it was found that fibre bundling increased with each cycle, suggesting the thf component was evaporating out of the small droplet (Fig. S7†). It is noted that the influence of solvation on gel formation is an area of current interest, with some significant progress being made relating a range of solubility parameters,^15^ and particularly Hansen solubility parameters,^16^ to gelation properties. It may be that the thf is selectively solvating the fibres, leading to inhibition of bundling, and only competes for inclusion in the calixarene cavity at higher concentrations. The kinetics of the competitive processes involved are unclear at this point, and require further investigation with a controlled environment to prevent solvent loss. *In situ* imaging of this nature will be vital to resolve if the gel structure changes over time, and may be useful to apply to other systems where kinetic effects are being investigated.^16^,^17^

## Conclusions

The assembly and disassembly of gel fibres formed by a low molecular weight gelator have been observed *in situ* using scanning probe microscopy for the first time. The results suggest that monitoring fibre assembly at a surface can be indicative of the behaviour in the bulk. Examining the impact of a gelation inhibitor showed that changes in gel fibre morphology and interactions can be readily observed, and will provide insight into the gelation process. Based on the results obtained to date, visualisation of fibre growth using this method may be useful in supporting, or overturning, expectations and orthodoxies in self-assembled gel science.

## Supplementary Material

Supplementary information

Crystal structure data
