# Supplementary material for: Investigating hydrogel formation using in situ variable-temperature scanning probe microscopy
Source: Chem Sci. 2015 Aug 3;6(11):6133–8. doi: 10.1039/c5sc02196k (PMC6054096; doi:10.1039/c5sc02196k)
Supplement: Supplementary file 1 [file SC-006-C5SC02196K-s001.pdf]

Electronic Supplementary Material (ESI) for Chemical Science.
This journal is © The Royal Society of Chemistry 2015

Investigating hydrogel formation using *in situ* variable-temperature scanning probe microscopy

Emily C. Barker, Ching Yong Goh, Franca Jones, Mauro Mocerino, Brian W. Skelton, Thomas Becker, and Mark I. Ogden

SI: Supporting Information

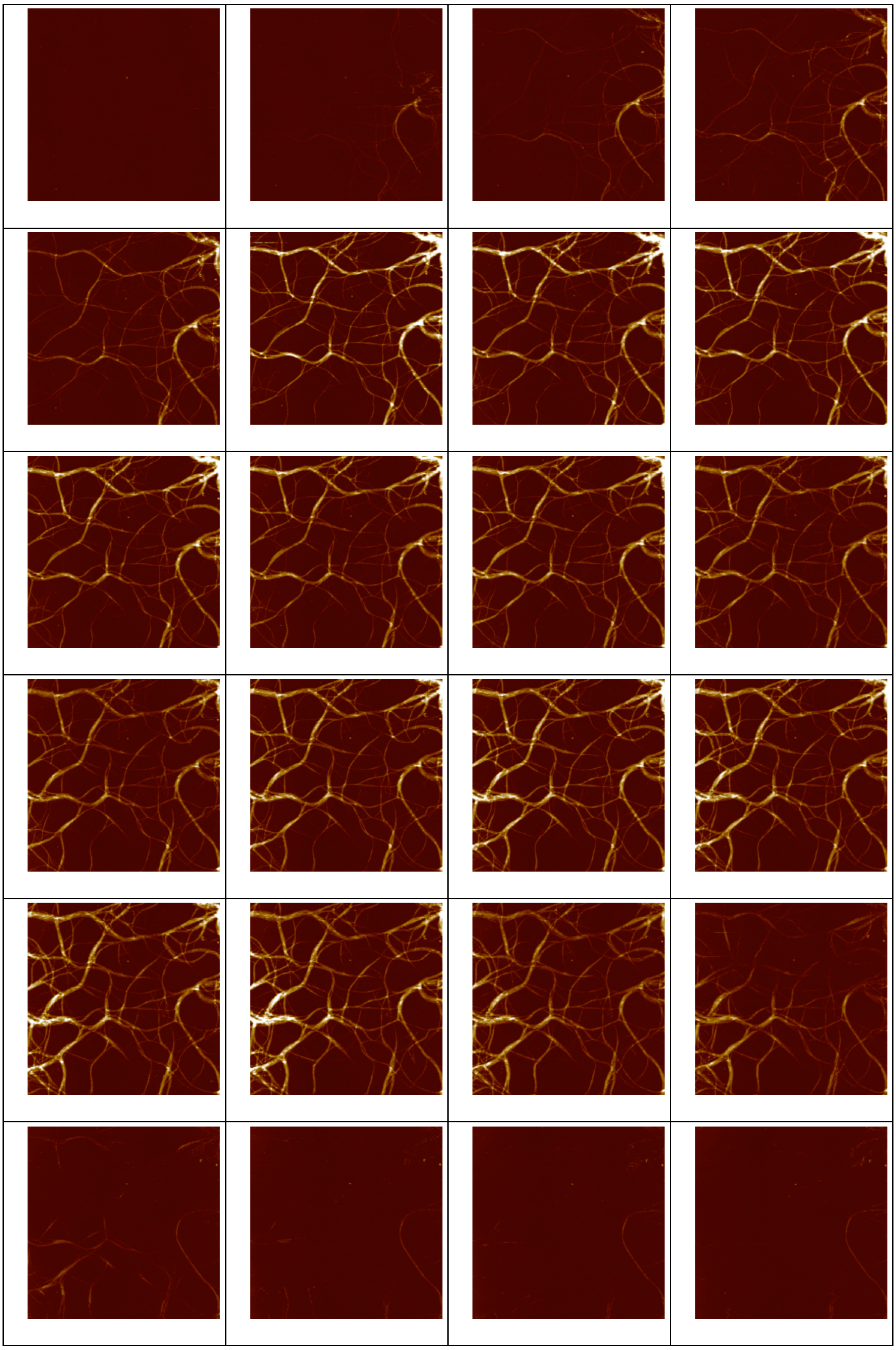

Figure S1(a). Scanning probe micrographs (topography data) of fibres formed by an aqueous mixture of **1** (25 mM) and $MgCl_2$ (20 mM) with changing temperature, imaged *in situ*. Scan size: 5 µm; z-scale: 30 nm; 4.4 min/image. Cross sectional analyses and temperature profile are given in Figure 3 of the manuscript.

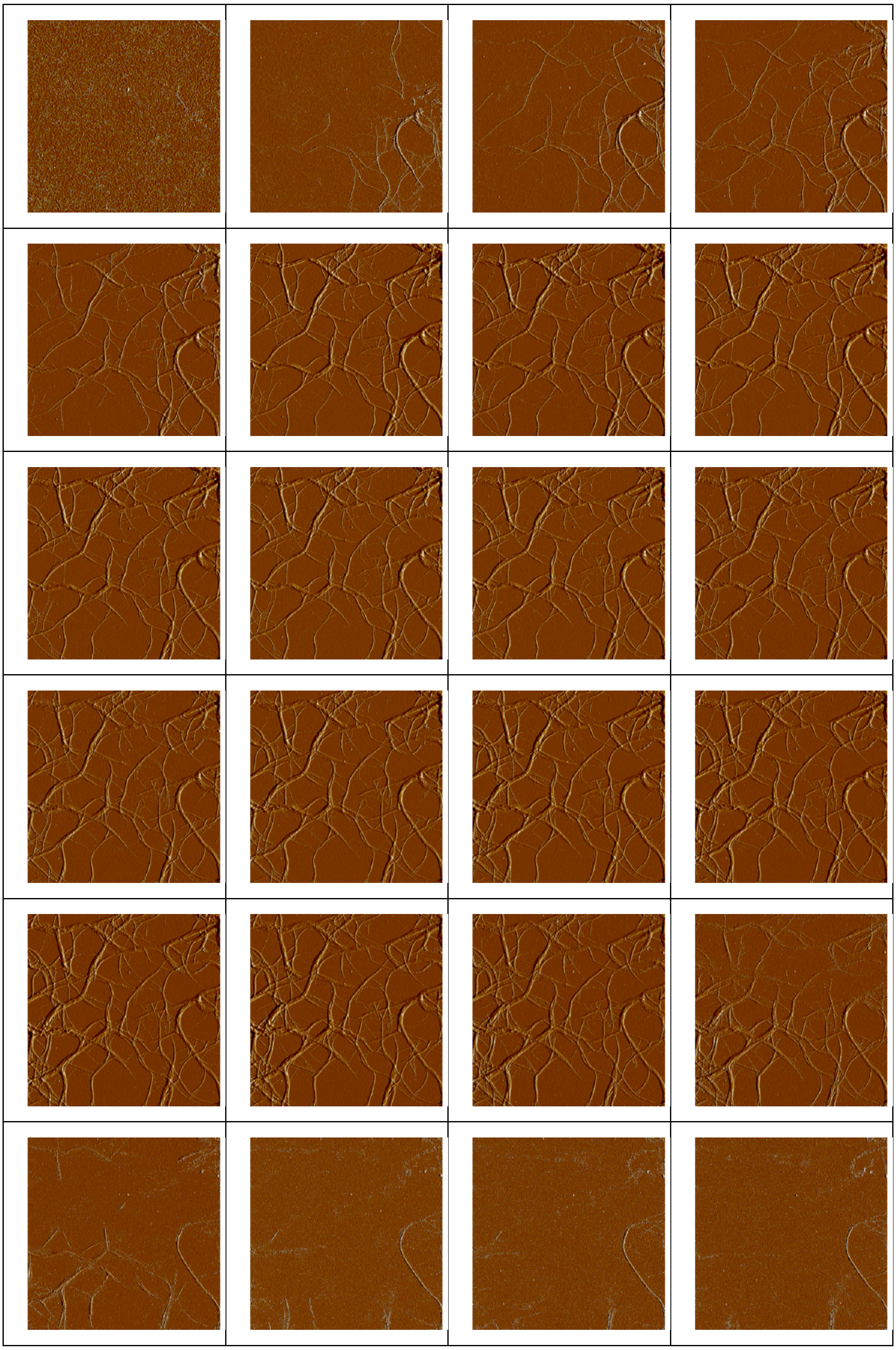

Figure S1(b). Scanning probe micrographs (PF error data) of fibres formed by an aqueous mixture of **1** (25 mM) and $MgCl_2$ (20 mM) with changing temperature, imaged *in situ*. Scan size: 5 µm; z-scale: 30 nm; 4.4 min/image. Cross sectional analyses and temperature profile are given in Figure 3 of the manuscript.

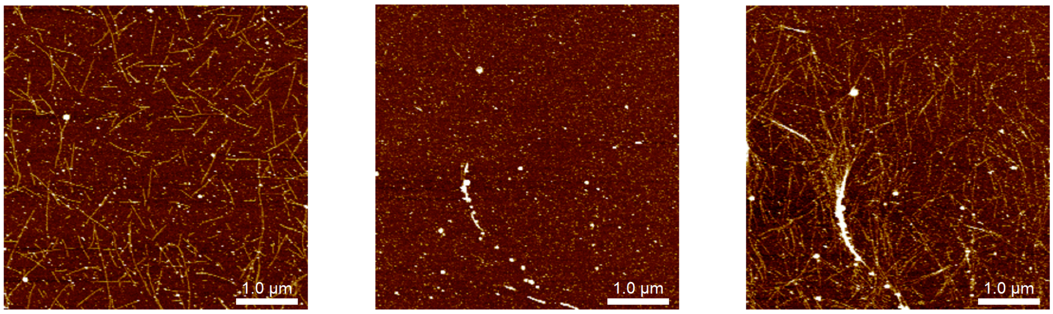

Figure S2. *In situ* atomic force micrographs of a **1** (20 mM) / $MgCl_2$ (25 mM) hydrogel, from left to right, initial deposition of gel fibres at 24 ˚C, disassembled gel imaged at 31 ˚C, reformed gel after cooling to 21 ˚C. There is no apparent correlation between the first and last images, suggesting that surface features of the substrate are not driving the assembly process.

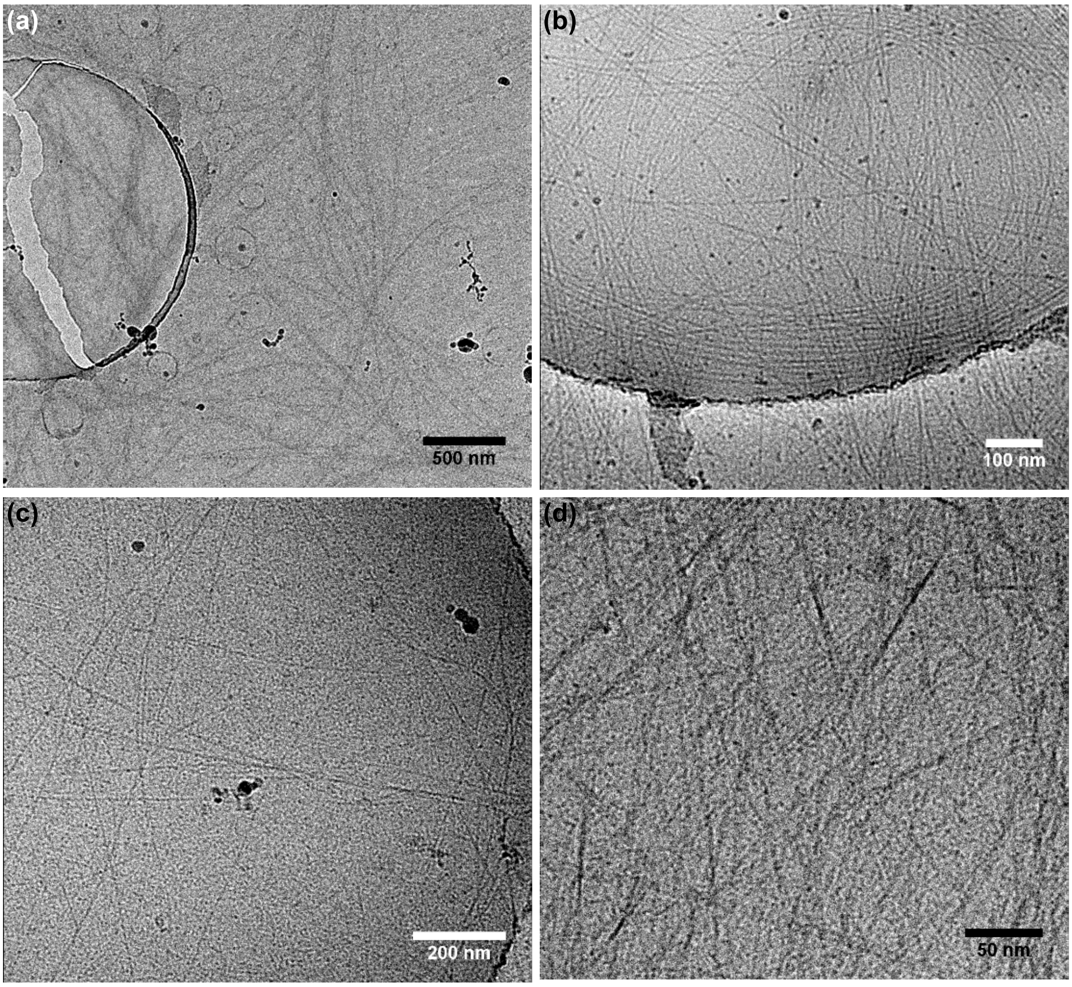

Figure S3. Cryo-TEM micrographs of calix[4]arene **1** (~20 mM) in the presence of (a) LiCl (20 mM), (b) NaBr (10 mM), (c) $CaCl_2$ (21 mM), and (d) $La(NO_3)_3$ (5 mM). All samples were blotted for 5 s (except **1**-LiCl sample, 30 s) at 5 °C. Dark round structures are crystalline ice.

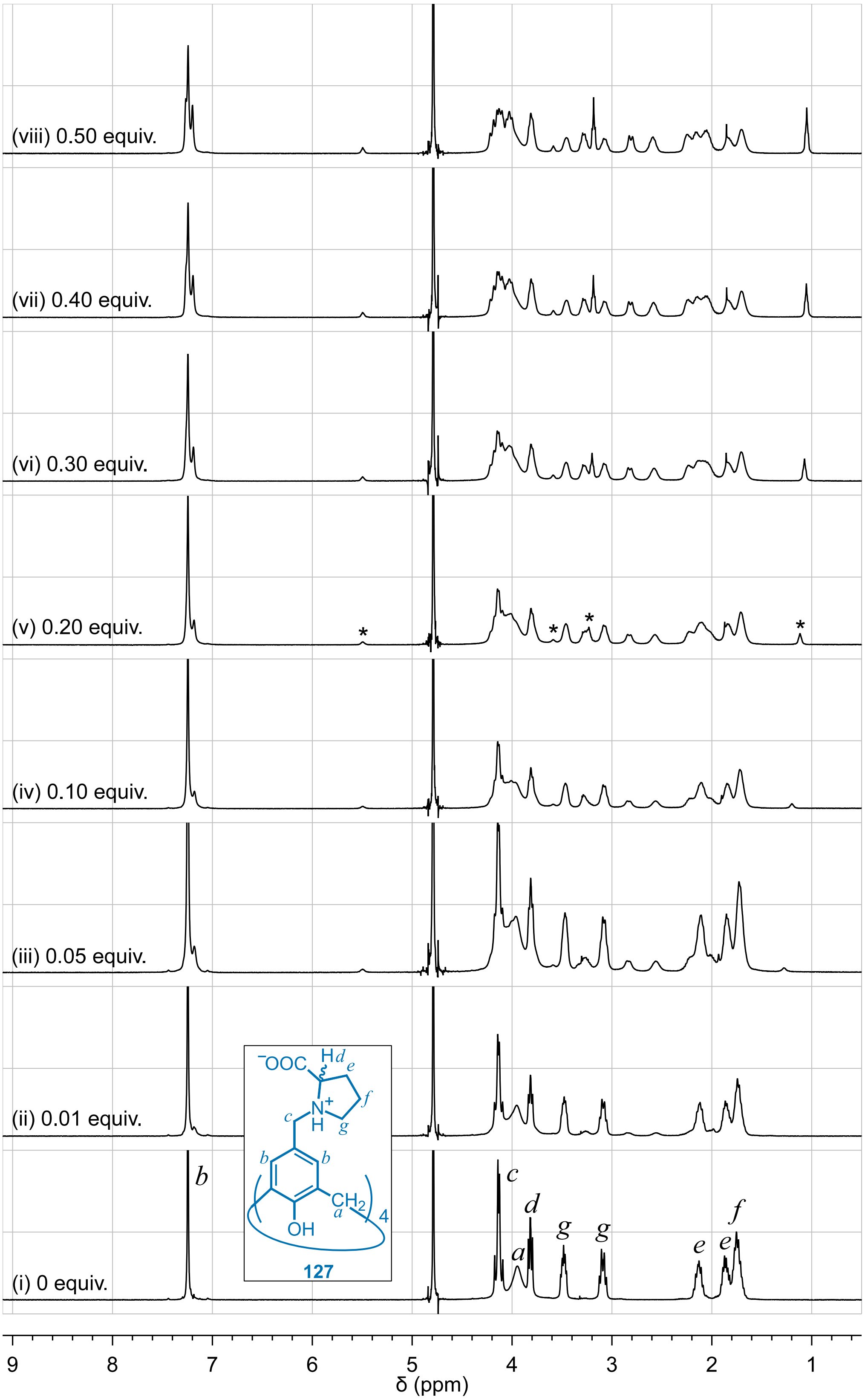

Figure S4. Titration of L-proline calix[4]arene **1** (20 mg mL^{-1} in D_2O) against THF (*). An initial solution of (i) **1** (20 mg mL^{-1}) in the presence of THF from (ii) 0.01 equiv. to (viii) 0.50 equiv. ^{1}H NMR spectra were acquired at 400 MHz.

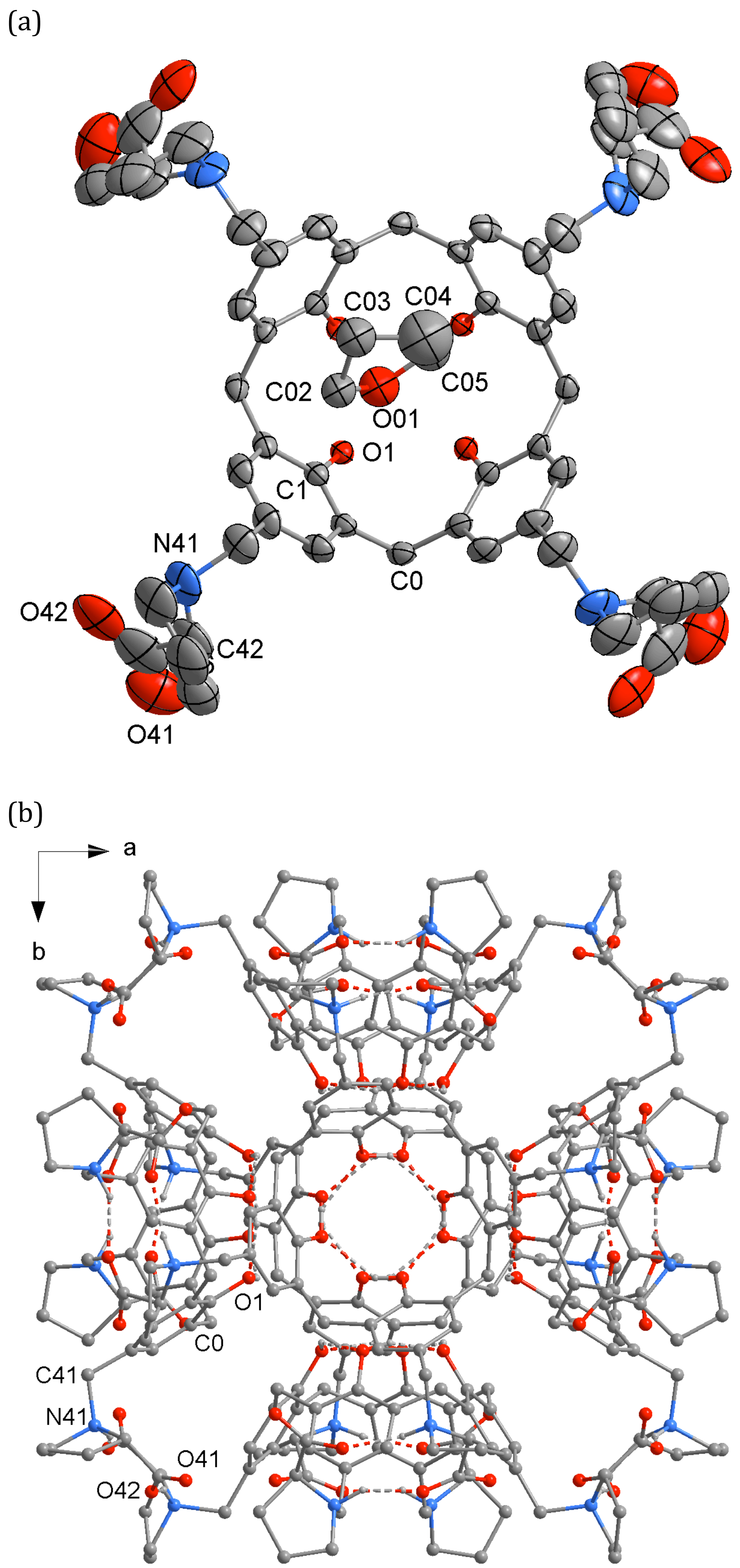

Figure S5. (a) Diagram of the thf solvate of **1**, and (b) the cubic hydrogen bonded assembly formed in the crystal viewed along the *c*-axis.

(a)

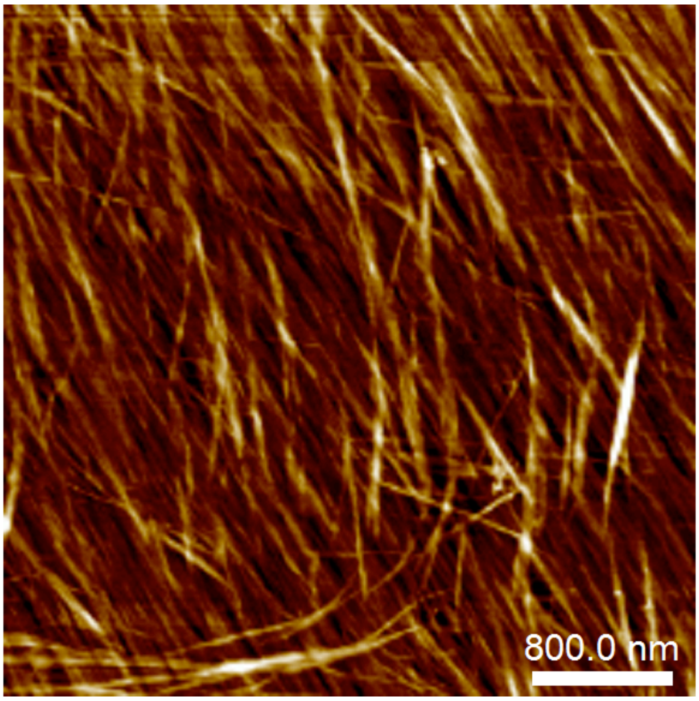

(b)

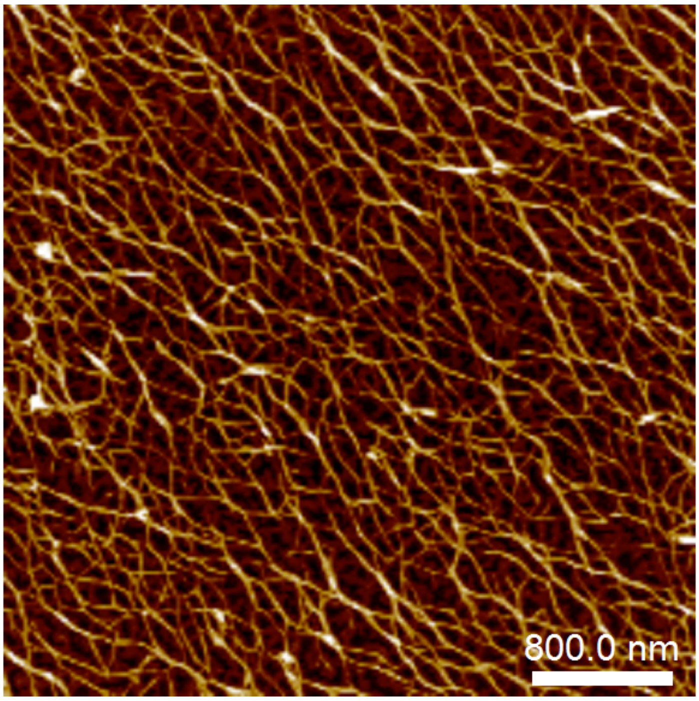

Figure S6. *Ex situ* atomic force micrographs of a **1** (25 mM)/$MgCl_2$ (20 mM) solution containing 0.6 % v/v tetrahydrofuran dropcast on mica. Images (a) and (b) are approximately 20 micron apart on the same sample, suggesting that evaporation of the thf is leading to variable restructuring of the fibres on the surface.

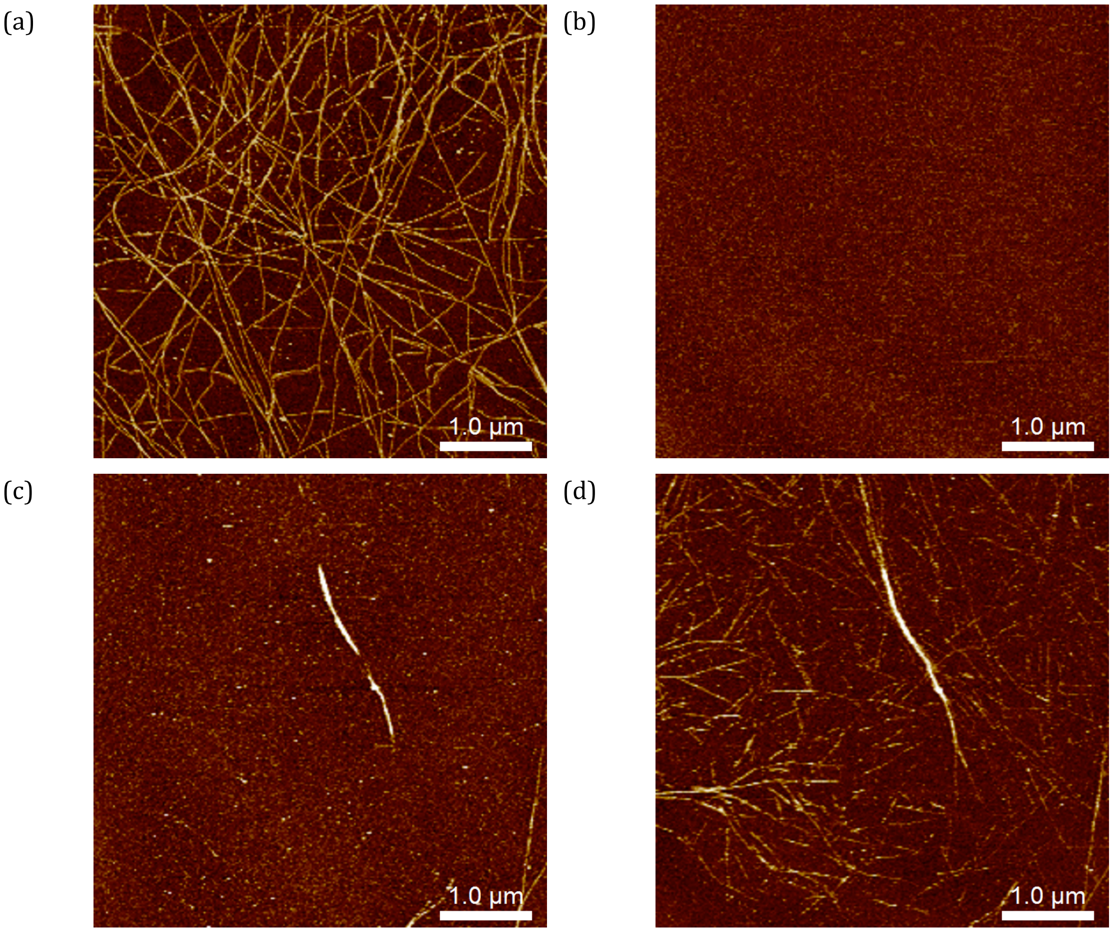

Figure S7. *In situ* atomic force micrographs of a **1** (25 mM)/$MgCl_2$ (20 mM) solution containing 0.6 % v/v tetrahydrofuran. The sequence of images show the fibres formed in the first cycle at 22 ˚C (a), removal of the fibres after heating to 30 ˚C (b), reformation of the fibres after cooling to 20 ˚C (c), and further cooling to 19 ˚C (d). The greater extent of fibre bundling observed after the heat/cool cycle is proposed to be caused by evaporation of the thf component.
